# Comparison of clinical course and outcome of acute pancreatitis according to the two main etiologies: alcohol and gallstone

**DOI:** 10.1186/s12876-015-0323-1

**Published:** 2015-07-25

**Authors:** Joon Hyun Cho, Tae Nyeun Kim, Sung Bum Kim

**Affiliations:** Division of Gastroenterology and Hepatology, Department of Internal Medicine, Yeungnam University College of Medicine, 317-1, Daemyung 5-dong, Nam-gu, Daegu, 705-717 Republic of Korea

**Keywords:** Acute pancreatitis, Etiology, Alcohol, Gallstone, Clinical course

## Abstract

**Background:**

Studies concerning clinical course and outcome of acute pancreatitis (AP) according to etiologies were rare, especially after year 2000. This study was designed to investigate the difference between the clinical course of alcoholic and biliary AP.

**Methods:**

Of the 153 patients diagnosed as AP with a first attack between January 2011 and January 2013, extensive clinical data of 50 patients with AP caused by alcohol and 76 patients with AP caused by gallstone were analyzed retrospectively. We compared the severity of AP defined by revised Atlanta classification in 2012, local complications, severity scores, and computed tomography severity index (CTSI) between alcoholic and biliary AP. We also evaluated the length of hospital stay, duration of NPO, and in-hospital mortality in each group.

**Results:**

Hemoglobin, hematocrit, and serum C-reactive protein level measured after admission for 24 h were significantly higher in the alcohol group than in the biliary group. Incidence of pseudocyst formation was significantly higher in the alcohol group than in the biliary group (20.0 % vs. 6.6 %, *P* = 0.023). Among prognostic scoring systems, only CTSI showed significant difference (*P* < 0.001) with a mean score of 3.0 ± 0.9 in the alcohol group and 1.7 ± 1.2 in the biliary group. Severe AP with organ failure persisting beyond 48 h was observed in 12 patients (24.0 %) in the alcohol group and one patient (1.3 %) in the biliary group (*P* < 0.001). There were 4 mortalities in the alcohol group only (*P* = 0.012).

**Conclusion:**

More severe forms of AP and local complication, such as pseudocyst formation, are associated with alcoholic AP compared with biliary AP.

## Background

Acute pancreatitis (AP) is a relatively common disease with incidence of 5 – 80 per 100,000 members of the population, although its prevalence varies in different countries and even in different areas of a given country, and the number of new cases has shown a steady increase in recent years [1–3]. Most patients with AP show spontaneous resolution without complications, however, 10 %-20 % of patients experience a severe attack with increased risk of mortality up to 25 % [4].

Gallstones and alcohol account for 70 %-80 % of all AP etiologies, although AP may be the consequence of numerous etiologic factors [5]. Identification and differentiation of etiologies of AP is important, because it can affect the specific therapeutic strategies, and eliminating causes of AP prevents further aggravation and recurrence of AP. Although many theories concerning the pathogenesis of AP caused by alcohol and gallstone have been proposed, no theory regarding the mechanisms for inducing inflammation of the pancreas in these two conditions has been widely accepted. However, the mechanisms of induction of AP by gallstones and alcohol appear to be different [6], which could lead to differences in clinical severity and rate of complications. Nevertheless, the question of whether the etiology is a risk factor for prognosis of AP remains controversial. There have been differing reports with regard to whether etiologic factors affect clinical outcome and mortality of AP. Various studies have reported that biliary AP is more severe and is associated with higher mortality than alcoholic AP [7–9]. A few investigators have reported higher complication and mortality in patients with alcoholic AP [10, 11], while some studies found no significant difference [12–14]. Most of these studies were reported before year 2000. Atlanta Classification [15] was revised in 2012, with an emphasis on persistent organ failure. In recent years, many advances, such as endoscopic sphincterotomy and radiologic intervention, have been made in treatment of AP. These might have changed clinical course and outcome of AP from the past.

This study was designed to investigate the role of the two main etiological factors, alcohol and gallstone, on the clinical course and outcome of AP.

## Methods

### Patients

Between January 2011 and January 2013, a total of 153 patients were diagnosed as AP with a first attack, and, among these patients, 126 patients with AP induced by two major causes, alcohol or gallstone, were included in this study. Extensive demographic, radiographic, and laboratory data of these consecutive patients were prospectively collected and analyzed retrospectively. Patients with AP on pre-existing chronic pancreatitis and previous history of AP were excluded from this study. This study was approved by the Institutional Review Board of Yeungnam University Hospital. Written informed consent was obtained from all patients.

All patients diagnosed as AP were treated with supportive care, including NPO, fluid resuscitation, and analgesics. Endoscopic retrograde cholangiopancreatography (ERCP) was considered in patients with biliary pancreatitis, and the timing of ERCP was dependent upon severity and clinical course of the patients. ERCP was performed as soon as possible in patients with concomitant acute cholangitis. We awaited spontaneous improvement of biliary obstruction for 24 – 48 hrs in patients with no associated cholangitis. Oral feeding was permitted when abdominal pain subsided and patients felt hunger sensation. Patients who remained asymptomatic with oral intake were discharged or underwent cholecystectomy if indicated.

### Definitions

AP was diagnosed if a patient had more than two of the three following findings: typical abdominal pain of AP (acute onset of a persistent and severe epigastric pain often radiating to the back), elevation of serum amylase and/or lipase levels above three times upper normal limit, and findings of imaging studies, including abdominal ultrasonography or computed tomography (CT), consistent with AP [15]. Alcohol was considered a cause of AP when patients had a history of alcohol consumption within 48 h before symptom onset and other possible causes were ruled out. Biliary AP was defined when a gallstone or biliary sludge was observed on abdominal ultrasonography or CT. The etiology was considered idiopathic when causative factors could not be identified from a detailed clinical and drug history or after initial investigations.

Severity of AP was determined according to the most recently revised Atlanta Classification. Mild AP was defined by the absence of organ failure and the absence of local or systemic complications. Moderately severe AP was defined by the presence of transient organ failure, local complications such as acute peripancreatic fluid collections (APFC) and acute necrotic collections (ANC), or exacerbation of co-morbid diseases. Severe AP was defined by persistent organ failure for more than 48 h. Organ failure was defined as a score of 2 or more for one of the three systems (respiratory, cardiovascular, and renal) using the modified Marshall scoring system [16].

Pancreatic pseudocyst was defined as an encapsulated fluid collection usually outside the pancreas with a well defined wall and homogeneous fluid density occurs more than 4 weeks after the onset of interstitial oedematous pancreatitis. Walled-off necrosis (WON) was defined as a mature and encapsulated collection of pancreatic and/or peripancreatic necrosis with a well defined wall and heterogeneous with liquid and non-liquid density, with varying degrees of loculations, occurs more than 4 weeks after the onset of necrotizing pancreatitis [15].

### Data collection

The following parameters were collected for each episode of AP: length of hospital stay, duration of NPO, in-hospital mortality, presence of organ failure, and local complications such as APFC, pseudocyst, ANC, and WON. Acute Physiology and Chronic Health Evaluation (APACHE)-II [17] and bedside index for severity in acute pancreatitis (BISAP) [18, 19] scores were calculated using data from the first 24 h after admission and the Ranson [20] score using data from the first 48 h. CT severity index (CTSI) [21] was calculated in patients who underwent contrast-enhanced CT (CECT) within 48 h after admission.

### Statistics

Data were collected in a Microsoft Excel database. After completion of data collection, the database was imported into SPSS for Windows (20.0, SPSS, Chicago, IL, USA). Continuous variables were expressed as mean with standard deviation (SD) and compared using Student’s *t*-test or Mann–Whitney test. Categorical variables were expressed as absolute numbers and proportions. Pearson’s χ^2^ test or Fisher’s exact test was used for comparison of categorical variables. A *P* value of < 0.05 was considered statistically significant.

## Results

### Etiology of AP

In a two-year time period, 153 patients diagnosed as AP with a first attack were admitted to our institution. The etiologies of AP were gallstone in 76 patients (49.7 %), alcohol in 50 patients (32.7 %), idiopathic in 24 patients (15.7 %), and malignancy in two patients, and hypertriglycedemia in one patient.

### Patient characteristics of alcoholic and biliary AP

Mean age of 126 patients with AP caused by two major causes, alcohol or gallstone, was 63.6 ± 15.4 years (range, 27 – 94) and 81 patients (64.3 %) were male (Table 1). Age and BMI did not differ significantly between the alcohol group and the biliary group (*P* = 0.104, and *P =* 0.798, respectively). Male was more predominant in the alcohol group than in the biliary group, with statistical significance (*P* < 0.001). Medical comorbidity did not differ significantly between the two groups. Initial laboratory findings of hemoglobin and hematocrit were significantly higher in patients with alcoholic AP compared to those with biliary AP (*P =* 0.002, and *P =* 0.011, respectively). Serum alanine aminotransferase level was significantly higher in the biliary group than in the alcohol group (*P* < 0.001). Serum C-reactive protein (CRP) level measured at admission (CRPi) did not differ significantly between the two groups, and serum CRP level measured after admission for 24 h (CRP_24_) was significantly higher in the alcohol group compared with the biliary group (*P* < 0.001). Serum amylase and lipase level were significantly higher in patients with biliary AP compared to those with alcoholic AP (*P* < 0.001, and *P =* 0.004, respectively). We compared serum pancreatic enzymes between two groups except two patients with renal failure in the biliary group.

**Table 1:** Baseline demographics and clinical characteristics of the patients

	Alcohol group (N = 50)	Biliary group (N = 76)	*P* value
Age, years			
Mean ± SD	52.3 ± 11.2	67.3 ± 14.9	0.104
Median (range)	51 (31–80)	71 (27–94)	
Male	41 (82.0)	40 (52.6)	<0.001
BMI (kg/m^2^)	22.7 ± 3.5	23.1 ± 3.5	0.798
Medical comorbidity			
Hypertension	8 (16.0)	21 (27.6)	0.129
Liver cirrhosis	2 (4.0)	1 (1.3)	0.334
Diabetes mellitus	6 (12.0)	9 (11.8)	0.978
Chronic kidney disease	0 (0)	2 (2.6)	0.413
Ischemic heart disease	2 (4.0)	4 (5.3)	0.744
Laboratory findings			
Hemoglobin (g/dL)	14.7 ± 3.1	13.2 ± 1.8	0.002
Hematocrit (%)	42.2 ± 7.6	38.5 ± 5.6	0.011
AST (IU/L)	229.4 ± 438.9	284.2 ± 317.1	0.499
ALT (IU/L)	98.5 ± 119.4	234.4 ± 226.3	<0.001
Creatinine (mg/dL)	1.2 ± 0.8	1.2 ± 0.8	0.758
LDH (IU/L)	973.6 ± 860.6	737.5 ± 425.6	0.078
CRPi (mg/dL)	8.6 ± 11.8	4.5 ± 6.3	0.106
CRP_24_ (mg/dL)	20.9 ± 10.1	7.7 ± 6.8	<0.001
Amylase (IU/L)	738.6 ± 648.4	2061.1 ± 1479.0	<0.001
Lipase (IU/L)	1090.7 ± 1036.8	2456.9 ± 2220.7	0.004

Values are expressed as the mean ± SD or n (%)

BMI, body mass index; AST, Aspartate aminotransferase; ALT, Alanine aminotransferase; LDH, Lactate dehydrogenase; CRPi, C-reactive protein measured at admission; CRP_24_, C-reactive protein measured after admission for 24 h

### Comparison of clinical course, outcome, and severity of alcoholic and biliary AP

Of the 50 patients with alcoholic AP, 12 patients (24.0 %) developed persistent organ failure for more than 48 h and were classified as severe AP according to the Atlanta Classification, and of the 76 patients with biliary AP, only one patient was classified as severe AP (Table 2). The number of patients with severe AP was significantly higher in the alcohol group than in the biliary group (*P* < 0.001). Thirty patients (60.0 %) in the alcohol group and 16 patients (21.1 %) in the biliary group developed moderately severe AP, respectively. Patients with moderately severe AP were more predominant in the alcohol group than in the biliary group, with statistical significance (*P* < 0.001). Eight patients (16.0 %) in the alcohol group and 59 patients (77.6 %) in the biliary group developed mild AP, respectively. The number of patients with mild AP was significantly higher in the biliary group than in the alcohol group (*P* < 0.001).

**Table 2:** Severity and local complications of alcoholic and biliary AP

	Alcohol group (n = 50)	Biliary group ( n = 76)	*P* value
Severity of AP			
Mild	8 (16)	59 (77.6)	<0.001
Moderately severe	30 (60)	16 (21.1)	<0.001
Severe	12 (24)	1 (1.3)	<0.001
Local complications			
APFC	15 (30)	14 (18.4)	0.131
Pseudocyst	10 (20)	5 (6.6)	0.023
ANC	3 (6)	3 (3.9)	0.597
WON	1(2)	0(0)	0.216

Values are expressed as the n (%)

AP, acute pancreatitis; APFC, acute peripancreatic fluid collection; ANC, acute necrotic collection; WON, walled-off necrosis

APFCs were observed on CECT in 15 of 50 patients (30.0 %) in the alcohol group and in 14 of 76 patients (18.4 %) in the biliary group (Table 2). In the early days of hospitalization, pancreatic and peripancreatic necrosis with ANC was noted in 3 patients (6 %) in the alcohol group and 3 patients (3.9 %) in the biliary group. Among these patients, 1 patient in alcohol group developed WON after 4 weeks from the onset of AP during the follow up period. No statistical differences in occurrence of APFC and ANC were observed between patients with alcoholic AP and those with biliary AP (*P* = 0.131 and, *P* = 0.597, respectively). Pseudocyst occurred significantly more often in patients with alcoholic AP (10/50, 20 %) than in those with biliary AP (5/76, 6.6 %) (*P* = 0.023).

No significant differences in Ranson, BISAP, and APACHE-II scores were observed between the alcohol group and the biliary group (3.1 ± 1.8 vs. 3.1 ± 1.3 (*P =* 0.861), 1.2 ± 1.0 vs. 1.2 ± 0.9 (*P* = 0.888), and 8.0 ± 5.7 vs. 6.8 ± 3.4 (*P =* 0.322), respectively) (Table 3). Mean CTSI in the alcohol group was 3.0 ± 0.9, compared with 1.7 ± 1.2 in the biliary group. Difference in scoring systems between the two groups was only noted in CTSI, with statistical significance (*P* < 0.001).

**Table 3:** Severity scores and CTSI in alcoholic and biliary AP

	Alcohol group (n = 50)	Biliary group (n = 76)	*P* value
Ranson score	3.1 ± 1.8	3.1 ± 1.3	0.861
BISAP score	1.2 ± 1.0	1.2 ± 0.9	0.888
APACHE-II score	8.0 ± 5.7	6.8 ± 3.4	0.322
CTSI	3.0 ± 0.9	1.7 ± 1.2	<0.001

Values are expressed as the mean ± SD

AP, acute pancreatitis; BISAP, bedside index of severity in acute pancreatitis; APACHE-II, Acute Physiology and Chronic Health Evaluation-II; CTSI, computed tomography severity index

No significant differences in hospital stay, duration of NPO, and the time to alleviation of symptoms were observed between the alcohol group and the biliary group (9.9 ± 5.2 vs. 7.9 ± 3.5 (*P* = 0.086), 5.4 ± 2.7 vs. 4.4 ± 2.0 (*P* = 0.068), and 3.1 ± 2.3 vs. 2.8 ± 1.7 (*P* = 0.539), respectively) (Table 4). Four mortalities (8.0 %) occurred during hospitalization in the alcohol group; No patient died in the biliary group.

**Table 4:** Clinical course and outcome in alcoholic and biliary AP

	Alcohol group (n = 50)	Biliary group n = 76)	*P* value
Hospital stay, days	9.9 ± 5.2	7.9 ± 3.5	0.086
NPO, days	5.4 ± 2.7	4.4 ± 2.0	0.068
Time to alleviation of symptoms, days	3.1 ± 2.3	2.8 ± 1.7	0.539
Mortality	4 (8.0)	0 (0)	0.012

Values are expressed as the mean ± SD or n (%)

AP, acute pancreatitis; NPO, nil per os

### Treatment of biliary AP

Of the 76 patients, 45 patients with no definite evidence of bile duct stone in imaging study showed spontaneous improvement without ERCP. Spontaneous passage of common bile duct (CBD) stone was suspected in these patients. Thirty one patients underwent early or delayed ERCP. Early ERCP within three days of admission was performed in 19 patients, and elective ERCP in 12 patients. Bile duct stone was identified in 23 patients, and stone removal with endoscopic sphincterotomy was performed.

## Discussion

AP has many causes and the proportion of each etiologic factor varies greatly in different countries. In general, the most frequent cause of AP is cholelithiasis, followed by alcohol. Together these etiologies are responsible for approximately 80 % of all episodes of AP [5]. In a previous study including 1,068 European patients with AP, the percentage of biliary AP varied between 24 and 71 %, and that of alcoholic AP between 6 and 60 % among different countries [13]. In our study, 50 % (76/153) of the patients were diagnosed with biliary AP and 33 % (50/153) with alcoholic AP, showing results similar to those of previous studies.

A number of studies have reported that alcoholic AP was more prevalent in men and younger patients [5, 22]. Our study showed that male was significantly predominant in the alcohol group compared with the biliary group, and patients with alcoholic AP were younger than those with biliary AP; however, this was not statistically significant.

Many studies have reported on the effect of etiologic factors on the clinical course and mortality of AP. A number of studies have reported that biliary AP has a more severe clinical course, complication, and mortality [7–9]. Some investigators have reported that patients with alcoholic AP have poorer prognosis compared to those with biliary AP [10, 11, 23] Other studies have reported that the clinical course, outcome, and mortality were not influenced by underlying etiology [12–14].

Some studies have reported on the difference of serum pancreatic enzymes between alcoholic AP and biliary AP. Serum amylase and lipase tend to be lower in alcoholic AP than in biliary AP [8, 9, 22]. Consistent with the previous studies, significantly lower serum amylase and lipase level was observed in alcoholic AP than in biliary AP. Serum markers such as CRP, lactate dehydrogenase (LDH), and hematocrit have been reported to be reliable parameters for early prediction of necrotizing pancreatitis [24, 25]. In this study, the CRP_24_, hemoglobin, and hematocrit were significantly higher in patients with alcoholic AP compared to biliary AP. Serum LDH level was also higher in the alcohol group than in the biliary group, although this was not statistically significant. These differences of laboratory findings may indicate that patients with alcoholic AP have a more severe clinical course and poorer prognosis compared to those with biliary AP. Our study showed that moderately severe and severe AP defined according to revised Atlanta Classification and the mortality were significantly more common in the alcohol group than in the biliary group, and the number of patients with mild AP was significantly lower in the alcohol group compared with the biliary group, although no differences were observed with regard to the scoring systems predicting the severity of AP, such as Ranson, BISAP, and APACHE-II scores. These results indicated that there was no difference in the severity of AP on admission between alcoholic and biliary AP, but the patients with alcoholic AP were more likely to become severe during the clinical course compared with biliary AP. In our study, duration of hospital stay and NPO tended to be longer in the alcohol group than in the biliary group.

Lower proportion of severe AP in the biliary group can be explained in part by the fact that progression of AP was likely to be prevented owing to early treatment of bile duct stone by ERCP. In general, many patients with biliary AP experience spontaneous passage of stone and ERCP is not recommended in these patients, while endoscopic removal of bile duct stone with ERCP is recommended if it does not pass spontaneously. The severity, clinical course and prognosis of biliary AP could be associated with concomitant acute cholangitis and cholestasis. In patients with acute cholangitis and ongoing biliary obstruction, urgent ERCP to remove bile duct stones may lessen the severity of biliary AP [26–29]. In our studies, 45 of 76 patients with biliary AP showed spontaneous improvement without ERCP, in whom spontaneous passage of CBD stone was suspected. Among 31 patients who underwent ERCP, 12 patients underwent elective ERCP after improvement of AP. Urgent (<24 h) or early (<72 h) ERCP [26, 30] was performed in 19 patients with poor response to medical therapy, and rapid improvement of pancreatitis was observed after endoscopic stone removal in these patients. Early recognition and removal of CBD stone by ERCP or spontaneous passage of the stones might have a beneficial influence on the clinical course of biliary AP. These timely interventions might contribute to change in clinical course and improvement of outcome in biliary AP, whereas patients with alcoholic AP could be treated only by supportive care such as NPO, aggressive fluid resuscitation, and close monitoring.

In the previous report, a tendency to have more peripancreatic infiltrations or fluid collections was observed for alcoholic AP compared with biliary AP [31]. In our study, frequency of pseudocyst was significantly higher in the alcohol group than in the biliary group, and CTSI in the alcohol group was significantly higher compared with that in the biliary group. Alcoholic AP usually occurs in patients with heavy alcohol intake for a long period of time, although single heavy drinking could lead to AP, and substantial damage to the pancreas has already occurred when AP occurs [32]. Underlying pancreatic ductal stricture and dilation due to longstanding pancreatic damage by alcohol are presumably present in patients with alcoholic AP, and, thus, they are more vulnerable to ductal disruption with subsequent leakage of pancreatic juice into the peripancreatic area and pseudocyst formation. On the other hand, biliary AP usually occurs from a single episode of bile duct obstruction caused by gallstone in relatively intact pancreas. This difference in duration of exposure to harmful impact may contribute to difference in disease severity between alcoholic and biliary AP. Lower incidence of walled-off necrosis in this study may be explained in part by lower incidence of pancreatic and/or peripancreatic necrosis at the time of admission. The reason of lower occurrence of pancreatic necrosis in this study was unclear, but multiple factors such as BMI, genetic and environmental factors might be associated.

There were some limitations in this study. First, although all patients in this study were enrolled prospectively over the study period, some clinical data, especially for the transferred patients, were missing due to lack of availability because the data of these patients were analyzed retrospectively. Second, the number of including patients was relatively small compared to other large scale clinical studies; therefore, comparison of some variables between two groups and further analysis was difficult. Nevertheless, this study was one of the few studies to compare clinical course and outcomes of AP caused by alcohol and gallstone especially in a non-Western area.

## Conclusion

In summary, results of our study demonstrated that more severe forms of AP and local complication, such as pseudocyst formation, are associated with alcoholic AP compared with biliary AP. The timely interventions such as endoscopic sphincterotomy and pathophysiologic difference may contribute to difference in disease severity between alcoholic and biliary AP.

## Abbreviations

AP: Acute pancreatitis
ERCP: Endoscopic retrograde cholangiopancreatography
CT: computed tomography
APFC: acute peripancreatic fluid collection
ANC: acute necrotic collection
WON: walled-off necrosis
APACHE: Acute Physiology and Chronic Health Evaluation
BISAP: bedside index for severity in acute pancreatitis
CTSI: Computed tomography severity index
CECT: contrast-enhanced computed tomography
SD: standard deviation
CRP: C-reactive protein
CRPi: C-reactive protein measured at admission
CRP_24_: C-reactive protein measured after admission for 24 h
CBD: common bile duct
LDH: lactate dehydrogenase
